# Editorial: Proteomics of Post-translational Modifications in Plants

**DOI:** 10.3389/fpls.2022.894314

**Published:** 2022-05-27

**Authors:** Pingfang Yang

**Affiliations:** State Key Laboratory of Biocatalysis and Enzyme Engineering, School of Life Sciences, Hubei University, Wuhan, China

**Keywords:** post-translation modification, phosphorylation, acetylation, glycosylation, malonylation, ubiquitylation

## Background

Compared with its corresponding genome, the proteome is more complex, which could be largely ascribed to the post-translational modifications (PTMs) that modify proteins. Currently, over 460 different PTMs have been reported (Vu et al., 2018), including phosphorylation, ubiquitylation, acetylation, etc., with many of them playing important physiological roles. As sessile organisms, plants are constantly exposed to various biotic or abiotic stresses that may threaten their survival. To cope with environmental stress, plants should be able to make an adjustment instantly, during which PTMs provide a faster reaction mechanism than protein synthesis. Of course, PTMs are also essential in long-term responses or basal developmental processes. Hence, study on PTMs as well as their implication on their target proteins' functions have been a major focus in plant proteomics.

## Phosphorylation

Phosphorylation is the most extensively studied modification. Because of its reversible feature, it is an ideal choice for proteins to execute transient functions without changing their abundance, which is important in the cascade of cellular signaling in nearly all biological processes. There are two well-characterized phytohormone signaling pathways, in which (de-)phosphorylation is known to be important. The first one is the ABA signaling pathway, which is repressed by PP2Cs through dephosphorylating and inactivating SnRK2s. Upon the perception of ABA by its receptor, SnRK2s are released from PP2C and phosphorylated, which could activate its kinase activity and then phosphorylate downstream transcription factors, such as ABI5 (Yang et al., 2017). The second one is the BR signaling pathway, in which the sequential phosphorylation of BRI1, BIN2, and BES1/BZR1 occurs (Guo et al., 2013). These two signaling pathways mediated by protein (de-)phosphorylation are very important for the regulation of seed germination, and have been well addressed in the article of Yu F. et al.. Except for the roles in phytohormone signaling, (de-)phosphorylation is also important in biotic and abiotic stress responses. Salinity is a serious abiotic stress that negatively affects plant growth and hence threatens the yield of crops worldwide (Zhao et al., 2020). Phosphoproteomic analyses on different plant responses to salinity stress have been widely conducted, which resulted in the identification of many conserved phosphorylation sites among different plants. However, changes of the phosphorylation intensity on these conserved sites may be different between germplasm showing different levels of resistance to stress. Phosphoproteomic analysis on two foxtail millet cultivars indicated the importance of phosphorylation of sucrose metabolic enzymes (Pan et al.). This strategy was also applied to study the pattern- and effector-triggered immunity (PTI and ETI) in tomato, which displayed both the commonly and distinctly occurring protein phosphorylation of PTI and ETI (Yu J. et al.).

## Acetylation and Other Lysine PTMS

Lysine acetylation (LysAc) was firstly identified in histones, which was shown to play important roles in epigenetics. Later, it was found to widely occur in many non-histone proteins, and regulate protein function in diverse manners (Philp et al., 2014). This modification is also reversible, which makes it another ideal regulatory strategy that has a transient effect on protein function during cellular signaling. Because of the advances in Lys-acetylated protein enrichment techniques and sensitivity of MS, LysAc proteomics studies have been widely conducted in many plant species, which substantially contribute to our understanding of the potential function of LysAc in physiological processes of plants. A LysAc proteomics study that focused on pepper cold resistance was conducted (Liu et al.). They indicated that cold temperature might inhibit the carbon assimilation process through affecting the acetylation status of the enzymes involved in this pathway (Liu et al.).

Except for acetylation, several other lysine modifications were also detected. Because of its importance in regulating protein destination, degradation, and cycling, ubiquitylation is an extensively studied modification. Ubiquitination was profiled in germinating rice seeds, which showed that ribosome subunit proteins were highly ubiquitinated (Yu F. et al., He et al., 2020). Malonylation of lysine is a relative new PTM, which plays regulatory roles in cell metabolism (Hirschey and Zhao, 2015). Studies focusing on this modification are still in their infancy in plants. A systematic analysis of lysine malonylation has been conducted in maize, which revealed that the enzymes involved in photosynthesis and the Calvin cycle were highly malonylated, indicating the potential crosstalk between the malonylation and acetylation (Xu et al.).

## Glycosylation

Protein glycosylation is very abundant in secretory proteins in eukaryotes. There are three types of glycosylation named N-, O-, and C-glycosylation, of which carbohydrates are linked to the amide group of asparagine residues (N-glycosylation), the hydroxyl group of serine, threonine, hydroxylysine, and hydroxyproline residues (O-glycosylation), and the carboxyl group of tryptophan residues (C-glycosylation) (Mendez-Yanez et al., 2021). Because the attached sites and the number of glycosyl structures can significantly vary, glycosylation might be the most complex PTM (Mendez-Yanez et al., 2021). Among them, N-linked glycans are widely observed at multiple sites on the majority of extracellular and transmembrane proteins, such as receptors and transporters, in plants (Strasser, 2016). Hence, enzymes catalyzing the linkage of glycosyl groups to suitable sites of proteins are physiologically important. The N-acetylglucosaminyltransferase II (GnTII), which catalyzes the transfer of N-GlcNAc residue from UDP-GlcNAc to N-glycan acceptor in Golgi, was found to be crucial for Arabidopsis to resist stresses (Yoo et al.).

## Outlook

Undoubtedly, PTMs play very important roles in many physiological processes. In the last decades, the great development of MS-based techniques has facilitated the identification of the proteome and its corresponding PTMs in an unprecedented large scale. However, there are still limitations in the following areas: (1) profiling the dynamic and site stoichiometry of PTMs; (2) crosstalk between different PTMs, especially those that co-exist at the same protein or different proteins of the same complex; and (3) function of different PTMs and the regulation on their target proteins' function. To discover these mysteries will be very helpful in elucidating the biological functions of PTMs.

## Author Contributions

The author confirms being the sole contributor of this work and has approved it for publication.

## Conflict of Interest

The author declares that the research was conducted in the absence of any commercial or financial relationships that could be construed as a potential conflict of interest.

## Publisher's Note

All claims expressed in this article are solely those of the authors and do not necessarily represent those of their affiliated organizations, or those of the publisher, the editors and the reviewers. Any product that may be evaluated in this article, or claim that may be made by its manufacturer, is not guaranteed or endorsed by the publisher.
